# Exploring factors predicting the effectiveness of oral semaglutide in Japanese individuals with type 2 diabetes switching from dipeptidyl peptidase 4 inhibitors: a pilot study

**DOI:** 10.3389/fcdhc.2025.1520389

**Published:** 2025-03-24

**Authors:** Takao Hirotsu, Kanta Taniguchi, Rimei Nishimura

**Affiliations:** 1 Department of Diabetes, Endocrinology and Hematology, Fuji Municipal Central Hospital, Fuji, Japan; 2 Department of Internal Medicine, Taniguchi Medical Clinic, Fujinomiya, Japan; 3 Division of Diabetes, Metabolism and Endocrinology, Department of Internal Medicine, Jikei University School of Medicine, Minato, Japan

**Keywords:** alcohol drinking, DPP-4 inhibitors, GLP-1 receptor agonists, type 2 diabetes, smoking

## Abstract

**Introduction:**

Oral semaglutide is a glucagon-like peptide-1 receptor agonist (GLP-1 RA) approved for the treatment of type 2 diabetes mellitus (T2DM). Findings from randomized controlled trials (RCTs) and real-world studies indicate that oral semaglutide leads to significant improvements in HbA1c and body weight, comparable to those observed with injectable GLP-1 RAs. Consequently, oral semaglutide is expected to significantly reduce barriers to initiating GLP-1 RA therapy in individuals with diabetes and may lead to an increased transition from dipeptidyl peptidase-4 inhibitors (DPP-4is) to GLP-1 RA therapy. This study was conducted to prospectively investigate the clinical characteristics predicting the achievement of HbA1c < 7% (52 mmol/mol) in Japanese individuals with T2DM who switched from DPP-4is to oral semaglutide.

**Methods:**

A total of 74 patients who switched from DPP-4is to oral semaglutide between December 2021 and October 2022 and completed the first 12-week therapy provided informed consent and were enrolled in the study. During the observation period, the oral semaglutide dose was uptitrated to achieve HbA1c < 7.0% (53 mmol/mol).

**Results:**

The study included a total of 44 individuals who achieved the target with oral semaglutide 3 mg (n=7), 7 mg (n=24), or 14 mg (n=13), and 17 individuals who did not (un-achieved group; n=17), based on their clinical characteristics and hematological findings. In the comparison between the Un-achieved group and the Achieved (3 to 14 mg) group, the proportions of “Current alcohol drinking (*p* = 0.030)” and “Current alcohol drinking and smoking (*p* = 0.029)” were higher in the Un-achieved group, whereas the proportion of “Taking 31 minutes or longer to have breakfast after drug administration (*p* = 0.022)” was higher in the Achieved (3 to 14 mg) group. A logistic regression analysis using the stepwise method identified “No current history of both smoking and alcohol drinking (0.083[0.014-0.485]; *p =* 0.006)” and “Taking 31 minutes or longer to eat breakfast after drug administration (0.117[0.029-0.480]; *p =* 0.003)” as factors predicting the achievement of the HbA1c < 7% (52 mmol/mol).

**Conclusion:**

Study findings suggest when considering switching T2D patients from DPP-4is to oral semaglutide, a detailed assessment of “current alcohol drinking and smoking status” and “the duration between the administration of oral semaglutide and breakfast” may be useful as a predictive indicator for achieving HbA1c < 7% (52 mmol/mol).

## Introduction

The goal of diabetes treatment is to improve metabolic dysfunction associated with hyperglycemia and to prevent the onset and progression of diabetic complications (i.e., microangiopathy and atherosclerotic diseases) in individuals with diabetes, thereby ensuring a life expectancy equivalent to that of healthy individuals and enabling them to lead a satisfactory life. Therefore, clinical practice guidelines for diabetes management generally recommend a treatment goal of HbA1c (hemoglobin A1c) < 7% (52 mmol/mol) to control diabetic complications (1). However, it has been suggested that far fewer than half of Japanese individuals with type 2 diabetes have achieved this target (2). In addition, treating individuals who have failed to achieve their targets with injectable antidiabetic drugs has been shown to be psychologically taxing for both patients and healthcare providers, and is likely to lead to clinical inertia, thereby hampering appropriate drug choice (3). Indeed, this is highly likely to account for the delays in initiating injectable glucagon-like peptide 1 receptor agonists (GLP-1 RAs), which are part of the current pharmacotherapy algorithm in people with obesity and diabetes mellitus. The frequency of use of GLP-1 RA use as a first-choice single drug has been reported to be less than 1% in these individuals (4). This contrasts with the increase in age-adjusted proportion of people with obesity, both men and women in all ages (32.6% and 19.9%, respectively) and of men and women aged 70 years or older (32.6% and 26.4%, respectively), as reported in the National Health/Nutrition Survey 2019 in Japan.

Meanwhile, among all individuals with type 2 diabetes treated with antidiabetic drugs, those treated with dipeptidyl peptidase 4 inhibitors (DPP-4is) account for 65.1%. This is due to the fact that impaired insulin secretion is an important contributor to the pathophysiology of type 2 diabetes in Japan, and that the majority of Japanese individuals with type 2 diabetes are elderly (5).

Against this background, the advent of the world’s first orally administered GLP-1 RA, semaglutide (oral semaglutide) is expected to significantly alleviate barriers to initiating GLP-1 RA therapy in individuals with diabetes, likely resulting in a switch from DPP-4is. Furthermore, among the phase III PIONEER studies of semaglutide conducted to date, the PIONEER 9 and 10 studies (6, 7) have demonstrated its the efficacy and safety in Japanese individuals with type 2 diabetes, suggesting a potential role for this drug in the management of type 2 diabetes. On the other hand, sodium N-(8-(2-hydroxybenzoyl amino)caprylate) (SNAC), which locally alters the pH in the stomach where the drug is dissolved, inhibits protein degradation and thus enhances the drug absorption (8); hence, it is important to take the drug with 120 mL of water on an empty stomach in the early morning, without consuming any food or drink 30 minutes before or after its dosing.

Evidence on factors predicting the efficacy of oral GLP-1 RAs remains limited. Therefore, this pilot study aimed to prospectively examine clinical characteristics and explore the potential presence of previously unidentified predictive factors associated with achieving HbA1c < 7% (52 mmol/mol) in Japanese individuals with type 2 diabetes who switched from DPP-4is to oral semaglutide in real-world settings.

## Methods

### Patients

Of all individuals treated in the outpatient setting at Fuji Municipal Central Hospital and Taniguchi Medical Clinic, Shizuoka, Japan, between December 2021 and October 2022, those who met the following criteria were included in the study: i) individuals aged ≥18 years; ii) those with a HbA1c value of ≥7% (52 mmol/mol) and <10.5% (90 mmol/mol); iii) those who had previously received DPP-4 inhibitors and were deemed by their treating physicians to require transition to a GLP-1 receptor agonist, subsequently initiated oral semaglutide at a starting dose of 3 mg, and, at an outpatient follow-up visit conducted at least 12 weeks after initiation, had no adherence- or tolerability-related barriers to continued treatment; iv) those who had an HbA1c measurement recorded at the initiation of oral semaglutide (12 weeks prior to study enrollment); v) those who provided written informed consent after completing the first 12 weeks of treatment with oral semaglutide, following a full explanation of the study; and vi) those who had neither been newly initiated on any drug other than oral semaglutide nor undergone any dose changes in their medication during the 12 weeks before providing informed consent.

Again, individuals who met the following criteria were excluded from the study: i) those with type 1 diabetes; ii) those receiving insulin therapy; iii) those who had been treated with a GLP-1 RA within 3 months prior to starting oral semaglutide 3 mg; iv) those with a history of severe ketoacidosis or diabetic coma in the past 6 months; v) women who were pregnant, likely to be pregnant, or breastfeeding; vi) those suspected of having diabetes due to other specific mechanisms or diseases; vii) those undergoing steroid treatment; viii) those with malignancy; ix) those with a severe infection or injury; and x) those deemed ineligible for the study by their attending physicians. The prospective observation period for evaluating treatment efficacy and dose uptitration in these enrolled patients was defined as February 2022 to January 2023.

### Study design

This was a prospective, observational study involving individuals who demonstrated good adherence and tolerance to oral semaglutide, without any adverse reactions such as gastrointestinal symptoms, during the uptitration to the maximum dose of 14 mg after switching from DPP-4is. Using medical records and a pre-distributed questionnaire, all patients who had been fully informed about the study and had provided informed consent were examined for their background characteristics (height, sex, and date of birth [age]), medical history (hypertension, dyslipidemia, and cardiovascular events), family history of diabetes, lifetime history of body weight (obesity), current history of smoking, current history of alcohol drinking, prior history of surgery for diabetic retinopathy, age at diabetes diagnosis, and time from oral semaglutide administration to breakfast.

Subsequently, individuals who achieved the HbA1c target of < 7% (52 mmol/mol) while receiving oral semaglutide at a dose of 3 mg were identified and included in the analysis. Those who met the target at this dose were categorized as the “3 mg achieved group,” based on measurements taken three months after initiation. Individuals who did not achieve the target continued with an increased dose of 7 mg. Similarly, those who met the target with 7 mg were categorized as the “7 mg achieved group.” If the target was not met, the dose was further increased to 14 mg. Individuals achieving the target with 14 mg constituted the “14 mg achieved group,” while those who did not meet the target were classified as the “un-achieved group,” based on measurements taken three months after treatment with semaglutide 14 mg.

Furthermore, data were collected from participants every 3 months on the following parameters: i) physical findings, i.e., body weight (body mass index [BMI]), systolic blood pressure, diastolic blood pressure, and waist circumference; ii) parameters for glycemic control, i.e., HbA1c and casual plasma glucose values; iii) general hematological test results, i.e., platelet counts; iv) general biochemical test results, i.e., liver function test results (AST, ALT, and FiB4-index), renal function test results, i.e., blood urea nitrogen (BUN) and creatinine (Cre) values and estimated glomerular filtration rates (eGFR), lipid test results, i.e., triglyceride (TG), high-density and low-density lipoprotein cholesterol (HDL-C and LDL-C) values, pancreatic endocrine test results, i.e., casual plasma C-peptide (CPR) values; and v) general urine test results, i.e., urinary albumin excretion rates and urinary protein excretion volumes.

A total of 74 patients who had switched from DPP-4is to oral semaglutide and completed the first 12 weeks of treatment at 3 mg as part of routine clinical care provided written informed consent and were enrolled in this prospective observational study.

**Figure 1:**
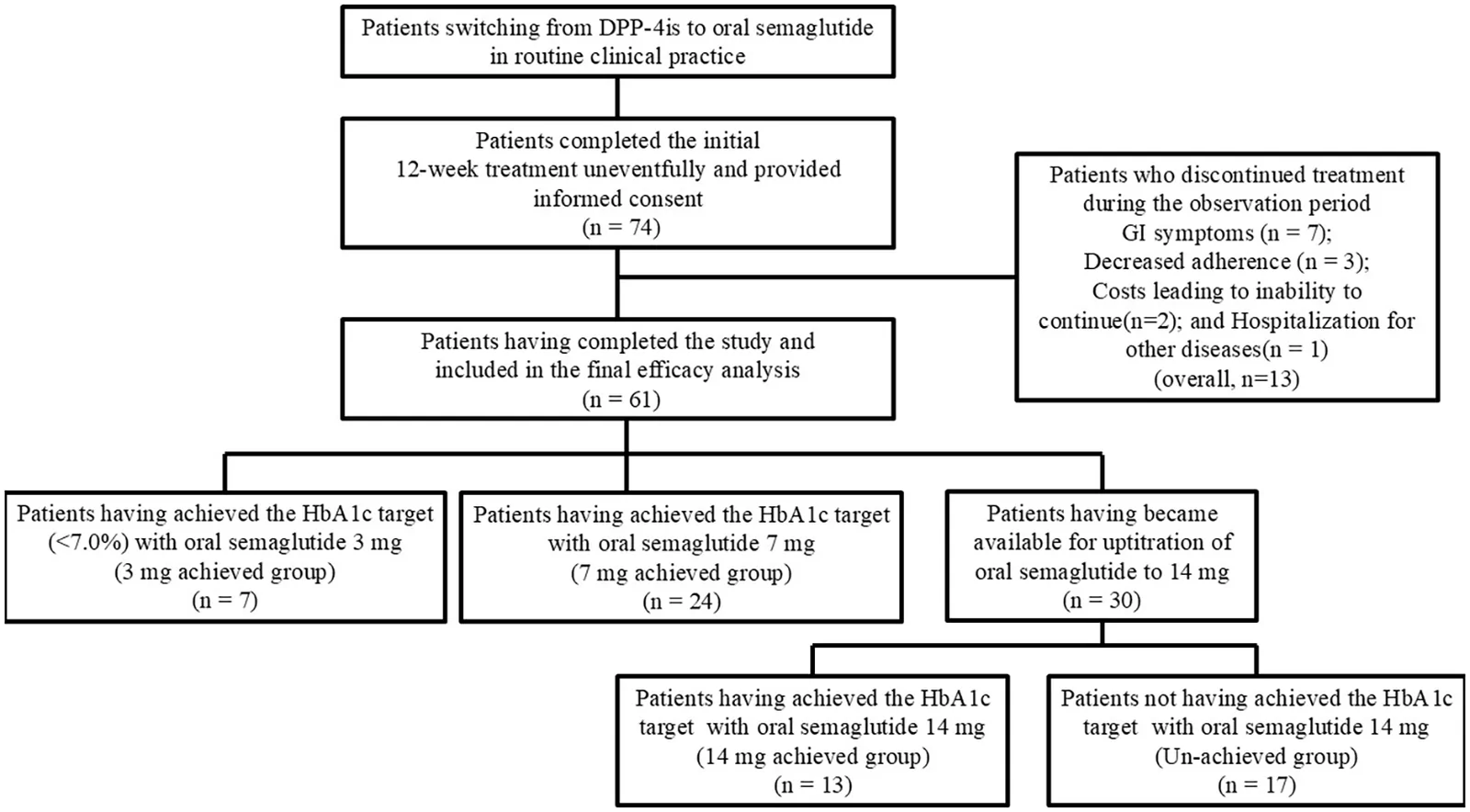
Participant flowchart in the present study. GI, gastrointestinal; HbA1c, hemoglobin A1c.

During the subsequent follow-up period, the dose of oral semaglutide was increased as needed to achieve an HbA1c level of < 7.0% (53 mmol/mol). Thirteen patients discontinued treatment due to gastrointestinal symptoms (n = 7), poor adherence (n = 3), hospitalization for conditions unrelated to diabetes (n = 1), and financial constraints (n = 2). The remaining 61 patients completed the study and were included in the efficacy analysis. Of these 61 patients, 7 achieved the HbA1c target of < 7.0% (53 mmol/mol) with 3 mg, 24 achieved the target with 7 mg, and 30 required dose escalation to 14 mg (**Figure 1**).

### Endpoints

The primary endpoints of the study included the baseline characteristics of the participants and the differences in hematological test results between those who achieved and those who did not achieve the HbA1c target (< 7% (52 mmol/mol)) while receiving oral semaglutide 14 mg (i.e., 14 mg achieved vs. unachieved groups), as well as those receiving varying doses of semaglutide (i.e., 3 to 14 mg achieved vs. unachieved groups).

As a secondary endpoint, changes in each hematological parameter were compared between the 14 mg achieved and unachieved groups.

### Statistical analysis

Statistical analysis was performed using SPSS (version 29.0.1.0). The Mann-Whitney test and chi-square test were used to compare individuals who achieved the HbA1c target (3 to 14 mg; achieved group) with those who did not (un-achieved group) in terms of their background characteristics and hematological test results. For comparisons among the three achieved groups (3 mg, 7 mg, and 14 mg), the Kruskal-Wallis test and chi-square test were applied. The Wilcoxon test was used to compare pre- and post-treatment values within the 14 mg achieved group and the un-achieved group. Additionally, the Mann-Whitney test was used to assess changes in each evaluated factor between the 14 mg achieved group and the un-achieved group. Continuous variables were presented as medians with interquartile ranges (IQRs), while categorical variables were expressed as counts and percentages.

Additionally, to assess the factors predicting achievement of HbA1c < 7% (52 mmol/mol) with oral semaglutide, multivariate logistic regression analyses were performed using both the forced entry method and the stepwise method for not only the 14 mg group but also the 3 to 14 mg group. Odds ratios (ORs) and 95% confidence intervals (CIs) were calculated for each predictor. To evaluate the model’s explanatory power, Nagelkerke R² was calculated. Statistical significance was set at *p* < 0.05.

## Results

### Baseline demographics

The baseline characteristics (prior to oral semaglutide administration) of the 61 individuals included in the analysis are summarized as follows (all continuous variables are shown as median [interquartile range]): men/women, 40/21; age, 61.0 (54.0–71.0) years; duration of diabetes, 15.0 (11.0–24.0) years; BMI, 27.3 (25.1–29.8) kg/m²; HbA1c, 7.9 (62) (7.6–8.7 (59–71)) % (mmol/mol); CPR, 3.0 (2.1–4.7) ng/mL; and eGFR, 69.0 (54.0–77.0) mL/min/1.73 m².

### Comparison of patients achieving and un-achieving HbA1c 7.0 with oral semaglutide

The 3 mg, 7 mg, and 14 mg achieved groups, as well as the un-achieved group, comprised 7, 24, 13, and 17 individuals, respectively (**Figure 1**). In the comparison between the un-achieved group and the 3 to 14 mg achieved group, the proportions of “current alcohol drinking (*p* = 0.030)” and “current alcohol drinking and smoking (*p* = 0.029)” were higher in the un-achieved group, whereas the proportion of “taking 31 minutes or longer to have breakfast after drug administration (*p* = 0.022)” was higher in the 3 to 14 mg achieved group. However, no significant differences were found between the groups in terms of baseline BMI and HbA1c values (*p* = 0.760 and *p* = 0.156, respectively). In the comparison among the three achieved groups (3 mg, 7 mg, and 14 mg), no significant differences were observed, except for a lower number of previously used oral hypoglycemic agents in the 3 mg group (*p* = 0.009) (**Table 1**).

**Table 1:** Clinical characteristics of patients achieving the HbA1c target (HbA1c < 7.0%: 3 to 14 mg, 3 mg, 7 mg, 14 mg achieved group, respectively) and failing to achieve the HbA1c target (HbA1c ≥ 7.0%: un-achieved group).

	Un-achieved group	3 to 14 mg achieved group	*P*-value	3 mg achieved group	7 mg achieved group	14 mg achieved group	*P*-value
No. of patients	17 (27.9%)	44 (72.1%)		7 (11.5%)	24 (39.3%)	13 (21.3%)	
Age (years)	59.0 (52.0–65.5)	64.0 (54.0–73.0)	0.184 ^+^	59.0 (52.0-68.0)	66.0 (54.0-73.0)	62.0 (54.5–71.0)	0.456 ^+++^
No. females	3 (18%)	18 (41%)	0.086^++^	1 (14%)	11 (46%)	6 (46%)	0.295^++^
No. of drugs previously used	4.0 (3.5–5.0)	4.0 (3.0–4.0)	0.197^++^	3.0 (1.0-3.0)	4.0 (3.0-5.0)	4.0 (3.0–5.0)	0.009^++^*
Duration of diabetes (years)	19.0 (13.0–26.5)	14.5 (10.0–21.8)	0.099 ^++^	12.0 (4.0-19.0)	15.0 (11.0-25.0)	13.0 (8.0–19.0)	0.435 ^++^
No. of patients on antihypertensive drugs	12 (70.6%)	33 (75.0%)	0.725 ^++^	7 (100%)	19 (79.2%)	7 (53.8%)	0.059 ^++^
No. of patients on antidyslipidemic drugs	15 (88.2%)	32 (72.8%)	0.197 ^++^	6 (85.7%)	15 (62.5%)	11 (84.6%)	0.248 ^++^
History of cardiovascular events	5 (29.4%)	13 (29.5%)	0.992 ^++^	2 (28.6%)	8 (33.3%)	3 (23.1%)	0.807 ^++^
Family history of diabetes	10 (58.8%)	26 (59.0%)	0.985 ^++^	5 (71.4%)	15 (62.5%)	6 (46.2%)	0.483 ^++^
History of obesity	15 (88.2%)	41 (93.0%)	0.528 ^++^	6 (85.7%)	24 (100%)	11 (84.6%)	0.144 ^++^
History of retinopathy	2 (11.8%)	10 (22.7%)	0.334 ^++^	1 (14.3%)	7 (29.2%)	2 (15.4%)	0.535 ^++^
Current smoking	10 (58.8%)	14 (31.8%)	0.053^++^	3 (42.9%)	8 (33.3%)	3 (23.1%)	0.645^++^
Current smoking (No alcohol drinking)	4 (23.5%)	9 (20.5%)	0.793^++^	2 (28.6%)	5 (20.8%)	2 (15.4%)	0.782^++^
Current alcohol drinking	11 (64.7%)	15 (34.1%)	0.030^++^*	3 (42.9%)	9 (37.5%)	3 (23.1%)	0.587 ^++^
Current alcohol drinking (No smoking)	5 (29.4%)	10 (22.7%)	0.587^++^	2 (28.6%)	6 (25.0%)	2 (15.4%)	0.739^++^
Current smoking + Current alcohol drinking	6(35.3%)	5(11.4%)	0.029^++^*	1(14.3%)	3(12.5%)	1(7.7%)	0.876^++^
Patients taking 31 minutes or longer to breakfast from drug administration	5 (29.4%)	29 (65.9%)	0.022^++^*	6 (85.7%)	14 (58.3%)	9 (69.2%)	0.387 ^++^
HbA1c (%)	8.4 (7.8–8.9)	7.9 (7.4–8.7)	0.156 ^+^	7.6 (7.2-7.8)	7.9 (7.4-8.8)	8.1 (7.6–8.8)	0.160 ^+++^
Body weight (kg)	79.2 (65.9–91.6)	71.7 (63.2–78.8)	0.106 ^+^	74.2 (66.0-78.0)	69.8 (63.2-79.2)	71.3 (61.0–81.0)	0.923 ^+++^
BMI (kg/m^2^)	27.3 (24.3–32.2)	27.4 (25.3–29.7)	0.760 ^+^	27.2 (26.7-29.5)	27.4 (24.6-29.3)	28.1 (25.3–30.3)	0.935 ^+++^
Waist circumference (cm)	97.0 (85.5–106.3)	96.3 (88.1–100.0)	0.987 ^+^	98.0 (90.0-101.0)	96.5 (86.5-100.0)	93.6 (88.0–102.0)	0.875 ^+++^
CPI	1.5 (0.9–2.5)	1.7 (1.3–2.5)	0.590 ^+^	2.5 (1.5-3.0)	1.7 (1.3-2.0)	1.5 (1.2–3.2)	0.580 ^+++^
CPR (ng/mL)	2.8 (2.1–5.3)	3.0 (2.1–4.6)	0.961 ^+^	3.5 (2.5-5.0)	3.0 (2.1-3.6)	2.6 (1.7–5.8)	0.484 ^+++^
CPG (mg/dL)	198 (166–244)	175 (145–225)	0.169 ^+^	161 (153-227)	172 (145-213)	180 (142–230)	0.954 ^+++^
eGFR (mL/min/1.73 m^2^)	68.0 (57.5–75.5)	70.0 (48.5–78.0)	0.936 ^+^	57.0 (30.0-80.0)	67.0 (44.5-83.0)	73.0 (65.0–78.0)	0.361 ^+++^
Urinary Alb (mg/gCr)	19.3 (8.6–88.9)	25.3 (13.3–82.9)	0.309 ^+^	79.6 (21.9-90.0)	26.1 (9.2-85.2)	21.1 (13.1–38.8)	0.148 ^+++^
FiB4-index	1.3 (1.0–1.8)	1.3 (1.0–2.0)	0.742 ^+^	1.2 (0.7-1.3)	1.3 (1.0-2.1)	1.3 (0.8–2.4)	0.297 ^+++^
AST (IU/L)	25 (19–33)	23 (19–30)	0.828 ^+^	21 (19-26)	22 (19-28)	29 (22–36)	0.179 ^+++^
ALT (IU/L)	27 (20–60)	23 (17–41)	0.359 ^+^	27 (13-35)	21 (17-31)	36 (16–69)	0.297 ^+++^
Platelet count (× 10^4^/μL)	22.4 (18.6–26.2)	23.3 (19.6–28.0)	0.440 ^+^	26.5 (19.1-28.8)	23.6 (20.0-27.8)	22.9 (19.6–25.0)	0.752 ^+++^

All data are presented as median (interquartile range) or number (%).

3 mg group: individuals confirmed to have achieved the HbA1c target (< 7%) with oral semaglutide 3 mg thus completing the study.

7 mg group: individuals confirmed to have achieved the HbA1c target (< 7%) with oral semaglutide 7 mg thus completing the study.

14 mg group: individuals confirmed to have achieved the HbA1c target (< 7%) with oral semaglutide 14 mg thus completing the study.

^+^ Mann-Whitney test, ^++^ chi-square test, ^+++^ Kruskal-Wallis test.

*<0.05.

Alb, albumin; ALT, alanine aminotransferase; AST, aspartate aminotransferase; BMI, body mass index; CPI, C-peptide index; CPR, C-peptide immune reactivity; CPG, casual plasma glucose; eGFR, estimated glomerular filtration rate; FiB4, fibrosis 4; HbA1c, hemoglobin A1c.

### Factors predicting achievement of HbA1c 7.0% with oral semaglutide

A logistic regression analysis (LRA) was performed using the forced entry and stepwise methods to identify factors predicting the achievement of HbA1c < 7% (52 mmol/mol). The analysis included eight variables: duration of diabetes in years, female sex, baseline BMI (kg/m²), no current history of alcohol drinking, no current history of smoking, baseline HbA1c value (%), taking 31 minutes or longer to have breakfast after drug administration, and no current history of both smoking and alcohol drinking as explanatory variables. The LRA using the forced entry method did not identify any factors predicting the likely achievement of HbA1c < 7.0% (52 mmol/mol) with oral semaglutide 14 mg. However, the LRA using the stepwise method identified two factors: “no current history of both smoking and alcohol drinking (0.042 [0.004-0.485]; *p* = 0.011)’ and “taking 31 minutes or longer to have breakfast after drug administration (0.086 [0.009-0.854]; *p* = 0.036)’ as predictors for achieving the HbA1c target (**Table 2**). The LRA was performed to explore factors predicting the achievement of the HbA1c target in the 3 mg, 7 mg, 14 mg achieved groups and the un-achieved group. The analysis using the forced entry method identified “taking 31 minutes or longer to have breakfast after drug administration (0.098 [0.019-0.506]; p = 0.006)” as a significant predictor, and “taking 31 minutes or longer to have breakfast after drug administration (0.117 [0.029-0.480]; *p* = 0.003)” as significant factors, consistent with the results summarized in **Table 3**. Thus, taken together, our study findings suggest that “no current history of both smoking and alchol drinking” and “taking 31 minutes or longer to have breakfast after drug administration” are the primary factors predicting the achievement of HbA1c < 7.0% (52 mmol/mol) when switching from DPP-4 is to oral semaglutide.

**Table 2:** Factors predicting the achievement of the HbA1c target (< 7.0%) after dosing with oral semaglutide 14 mg.

Variable	Model 1^+^		Model 2^++^	
	Odds ratio	*P*-value	Odds ratio	*P*-value
Duration of diabetes (years)	1.111 (0.926-1.333)	0.258		
Female (vs. male)	0.885 (0.073–10.744)	0.924		
BMI (kg/m^2^)	1.184 (0.816–1.718)	0.374		
No alcohol drinking (vs. alcohol drinking)	2.174 (0.044–108.032)	0.697		
No smoking (vs. smoking)	0.350 (0.009–14.234)	0.579		
No alcohol drinking and No smoking	0.034 (0.000-8.117)	0.226	0.042 (0.004-0.485)	0.011^*^
HbA1c (%)	0.684 (0.138–3.390)	0.641		
Time ≥ 31 minutes	0.083 (0.005-1.286)	0.075	0.086 (0.009-0.854)	0.036*

^+^forced entry method; ^++^stepwise method.

*<0.05.

BMI, body mass index; HbA1c, hemoglobin A1c.

Time>31 minutes: Patients taking 31 minutes or longer to have breakfast after drug administration.

Nagelkerke R² for Model 1and Model 2 are 0.600 and 0.519, respectively.

(Logistic regression model).

**Table 3:** Factors predicting the achievement of the HbA1c target (< 7.0%) after dosing with oral semaglutide (3 to 14 mg).

Variable	Model 1^+^		Model 2^++^	
	Odds ratio	*P*-value	Odds ratio	*P*-value
Duration of diabetes (years)	1.062 (0.969–1.164)	0.197		
Female (vs. male)	0.738 (0.110–4.957)	0.755		
BMI (kg/m^2^)	1.103 (0.891–1.366)	0.367		
No alcohol drinking (vs. alcohol drinking)	0.613 (0.061–6.164)	0.678		
No smoking (vs. smoking)	0.391 (0.044–3.456)	0.398		
No alcohol drinking and No smoking	0.177 (0.007-4.552)	0.296	0.083 (0.014-0.485)	0.006*
HbA1c (%)	1.713 (0.631–4.649)	0.290		
Time ≥ 31 minutes	0.098 (0.019-0.506)	0.006*	0.117 (0.029-0.480)	0.003*

^+^forced entry method; ^++^stepwise method.

*<0.05.

BMI, body mass index; HbA1c, hemoglobin A1c.

Time>31 minutes: Patients taking 31 minutes or longer to have breakfast after drug administration.

Nagelkerke R² for Model 1 and Model 2 are 0.460 and 0.355, respectively.

(Logistic regression model).

Furthermore, a comparison of changes in the parameters evaluated while on oral semaglutide showed that the 14 mg achieved group had a significantly larger change in waist circumference compared to the un-achieved group (-5.5 cm [-8.3 – -3.6 cm] vs. -2.8 cm [-6.0 – 0.5 cm]; p = 0.039) (**Table 4**).

**Table 4:** Change in parameters evaluated in patients achieving and not achieving HbA1c < 7.0% (achieved and un-achieved groups) after dosing with oral semglutide 14 mg.

	Achieved group				Un-achieved group				*P*-value^++^ (change)
	Baseline	Post-dose	Change	*P*-value^+^	Baseline	Post-dose	Change	*P*-value^+^	
HbA1c (%)	8.1 (7.6–8.8)	6.7 (6.5–6.9)	-1.3 (-2.2 – -0.8)	< 0.001*	8.4 (7.8–8.9)	7.4 (7.1–7.7)	-0.6 (-1.5 – -0.2)	0.018*	0.024*
Body weight (kg)	71.3 (61.0–81.0)	66.8 (57.5–75.1)	-5.4 (-6.4 – -3.0)	< 0.001*	79.2 (65.9–91.6)	77.1 (60.5–86.7)	-4.1 (-5.9 – -2.3)	< 0.001*	0.295
BMI (kg/m^2^)	28.1 (25.3–30.3)	27.3 (23.0–28.0)	-1.8 (-2.7 – -1.1)	< 0.001*	27.3 (24.3–32.2)	26.5 (22.3–30.4)	-1.4 (-1.9 – -0.8)	< 0.001*	0.116
Waist circumference (cm)	93.6 (88.0–102.0)	89.3 (81.5–97.0)	-5.5 (-8.3 – -3.6)	0.003*	97.0 (85.5–106.3)	93.3 (81.4–103.0)	-2.8 (-6.0– 0.5)	0.020*	0.039*
CPI	1.5 (1.2–3.2)	2.3 (1.6–4.7)	0.6 (0.2–2.5)	0.013*	1.5 (0.9–2.5)	2.1 (1.5–2.6)	0.3 (-0.3–0.9)	0.093	0.161
CPR (ng/mL)	2.6 (1.7–5.8)	2.8 (2.2–5.6)	0.2 (-0.8–1.4)	0.463	2.8 (2.1–5.3)	3.2 (2.1–4.7)	0.1 (-0.8–1.6)	0.776	0.802
CPG (mg/dL)	180 (142–230)	112 (106–131)	-52 (-119– -22)	0.002*	198 (166–244)	155 (134-190)	-21 (-67–29)	0.163	0.126
eGFR (mL/min/1.73 m^2^)	73.0 (65.0–78.0)	74.0 (65.0–83.5)	1.0 (-2.0–5.0)	0.326	68.0 (57.5–75.5)	68.0 (59.0–80.5)	1.0 (-1.0–7.5)	0.201	0.753
Urinary Alb (mg/gCr)	21.1 (13.1–38.8)	27.0 (15.0–34.3)	3.0 (-6.2–21.0)	0.209	19.3 (8.6–88.9)	17.3 (9.0–86.8)	1.3 (-7.6–9.0)	0.796	0.516
FiB4-index	1.3 (0.8–2.4)	1.3 (0.8–1.4)	0.0 (-0.4–0.1)	0.263	1.3 (1.0–1.8)	1.2 (0.8–1.6)	0.0 (-0.3–0.2)	0.523	0.586
AST (IU/L)	29 (22–36)	21 (18–29)	-4 (-18–0)	0.030*	25 (19–33)	21 (18–27)	-2 (-9–2)	0.114	0.346
ALT (IU/L)	36 (16–69)	27 (19–44)	-10 (-28–4)	0.037*	27 (20–60)	23 (18–45)	-4 (-12–2)	0.049*	0.571
Platelet count (× 10^4^/μL)	22.9 (19.6–25.0)	23.4 (20.2–25.1)	0.5 (-2.9–1.9)	0.807	22.4. (18.6.–26.2)	22.4 (19.8.–25.9)	0.5 (-0.2–1.3)	0.167	0.630

Data are presented as median (interquartile range).

^+^ Wilcoxon-test ^++^ Mann-Whitney test.

*<0.05.

Alb, albumin; ALT, alanine aminotransferase; AST, aspartate aminotransferase; BMI, body mass index; CPI, C-peptide index; CPR, C-peptide immune reactivity; CPG, casual plasma glucose; eGFR, estimated glomerular filtration rate; FiB4, fibrosis 4; HbA1c, hemoglobin A1c.

## Discussion

This prospective, observational study was conducted in 61 individuals who were treated with oral semaglutide 3 to 14 mg. It compared those who achieved the HbA1c target (n = 44) with those who failed to achieve the HbA1c target (n = 17). LRA identified “no current history of both smoking and alcohol drinking” and “taking 31 minutes or longer to have breakfast after drug administration’ as factors predicting the achievement of the HbA1c target following dosing with oral semaglutide (3 to 14 mg).

It has been shown that GLP-1 receptor agonists exert strong, multifaceted protective effects against diabetic vascular complications beyond glycemic control. In vascular endothelial cells, they reduce the expression of TNF-α, decrease the production of ROS, inhibit macrophage adhesion and activation, and protect against microvascular injury (9). Additionally, by increasing cAMP levels in cardiomyocytes, GLP-1 RAs have been shown to provide cardioprotective effects (10). In fact, the large-scale clinical trial SUSTAIN-6, which used the injectable GLP-1 receptor agonist semaglutide, reported improvements in non-fatal stroke and renal outcomes (11). Although not a large-scale trial, it has been reported that oral semaglutide is effective in improving outcomes for patients with diabetic kidney disease (DKD) (12). Furthermore, the SURPASS-4 trial with the GIP/GLP-1 receptor agonist tirzepatide reported a 41% reduction in the risk of composite renal outcomes and a 59% reduction in the risk of new-onset macroalbuminuria compared to insulin glargine (13).

The finding that “taking 31 minutes or longer to have breakfast after drug administration’ may predict the efficacy of oral semaglutide aligns with previous reports suggesting that prolonged fasting after administration increases the bioavailability of oral semaglutide (approximately 0.8% at 30 minutes, reaching a plateau of around 1.4% at 120 minutes) (14). The absorption of oral semaglutide is inhibited by the presence of food or large amounts of liquid in the stomach (15). Therefore, counseling patients to maintain a fasting period of at least 30 minutes after administration, while also monitoring the amount of water consumed during administration, is crucial for optimizing the efficacy of oral semaglutide.

This study also demonstrated that a “current history of both smoking and alcohol drinking” is a predictor of the efficacy of oral semaglutide. Oral drug delivery can be challenging due to various obstacles presented by the gastrointestinal (GI) tract, including complex pH environments, digestive enzymes, mucus barriers, and epithelial permeability (15). SNAC increases drug absorption in the stomach through several mechanisms. First, SNAC acts as a localized buffer to neutralize the pH of the microenvironment surrounding the semaglutide tablet, stabilizing semaglutide when exposed to gastric fluids and protecting it from degradation by gastric enzymes (8). Second, SNAC reduces the oligomerization of semaglutide, which could affect absorption (8). Finally, SNAC interacts with and fluidizes lipid membranes, thus increasing their permeability and enhancing the transcellular passage of semaglutide (8). The pharmacokinetics of oral semaglutide in patients with type 2 diabetes and upper gastrointestinal disorders (chronic gastritis and/or gastroesophageal reflux disease) were evaluated in a Phase 1, open-label, parallel-group study, and no significant differences were observed (16). On the other hand, a previous study evaluating the effects of oral semaglutide alone or in combination with omeprazole demonstrated increased exposure to semaglutide with the combination regimen compared to semaglutide alone, although the difference was not statistically significant (17).

It has been reported that chronic alcohol consumption leads to delays in gastric emptying and small-intestinal transit time (18). While nitric oxide (NO) is known to be involved in the regulation of gastrointestinal motility and is synthesized by neuronal nitric oxide synthase (nNOS), chronic alcohol consumption has been shown to decrease the expression of nNOS, thereby impairing gastrointestinal motility. Additionally, it has been demonstrated that in individuals with alcohol intake, vagal afferent neurons mediate the inhibitory effect of ethanol on gastrointestinal motility (19). For these reasons, it is likely that oral semaglutide exerted suboptimal effects in individuals with a history of alcohol drinking, as they may have had excess food residue that destabilized drug absorption. Of interest is a report suggesting the role of GLP-1 RAs in improving alcohol-induced impairment of gastric mucosal blood flow via NO/calcitonin gene-related peptide (CGRP) receptors (20). In the glomerulus, it has been reported that the administration of GLP-1 receptor agonists inhibits the Ang II signaling pathway through phosphorylated c-Raf (Ser338) via phosphorylated c-Raf (Ser259), thereby exerting a protective effect on endothelial cells (21). A similar mechanism may also inhibit the decrease in nNOS expression associated with alcohol consumption in the gastrointestinal tract, potentially improving peristaltic movement. Currently, no studies have directly investigated whether alcohol consumption affects the efficacy of GLP-1 receptor agonists. However, several reports have explored the association between GLP-1 receptor agonists and alcohol consumption. In a secondary analysis of RCT participants taking dulaglutide, compared to placebo, participants were 29% more likely to reduce alcohol intake (relative effect size 0.71, 95% CI 0.52-0.97, *p* = 0.04) (22). Observational studies showed fewer alcohol-related healthcare events and a significant reduction in alcohol use with GLP-1 RA treatment compared to DPP-4is use, no treatment, and/or alcohol intake at baseline (23).

The effects of smoking on the gut may be partly attributed to the large amounts of particulate matter inhaled by smokers. The nicotine concentration in gastric juice is reported to be 10 times higher than in arterial blood and 80 times higher than in venous blood (24). Nicotine can induce gastroesophageal reflux disease by blocking cholinergic receptors, leading to a decrease in lower esophageal pressure (25, 26). However, the gut may also be impacted by circulating components. Additionally, smoking reduces the salivary secretion rate and decreases the concentration of bicarbonate in saliva, thereby prolonging acid clearance time (27). Evidence suggests that chronic smoking (in individuals who have smoked for more than two years) may increase gastric acid secretion and lower gastric pH (28). Consistent with this, several studies have shown a positive correlation between smoking and the likelihood of Helicobacter pylori infection and disease progression (29). Furthermore, chronic smoking appears to alter mucus production in both the gastric (28) and intestinal mucosa (30), as well as impair intestinal mucosal repair (28). The vasoconstrictive and procoagulant effects of cigarette smoke may also influence the gut. For instance, chronic smoking has been reported to alter microcirculation and significantly reduce blood flow to the gut mucosa (31). Similar to alcohol consumption, no studies have directly investigated whether smoking affects the efficacy of GLP-1 RAs. However, recent research indicates that GLP-1 RAs reduce voluntary nicotine intake and seeking behaviors, and prevent withdrawal-induced hyperphagia and weight gain (32, 33). Emerging evidence also suggests that GLP-1 RAs improve cognitive deficits, as well as depressive- and anxiety-like behaviors, which contribute to smoking relapse during withdrawal (34).

The habits of smoking and alcohol consumption often co-occur (35). Previous epidemiological studies have reported that the concurrent use of smoking and alcohol is associated with an increased risk of cancer, neurocognitive disorders, and mortality (36–40). In the context of diabetes, a study conducted in Korea compared the triglyceride-glucose index (TyG), a simple, rapid, and cost-effective indicator of insulin resistance that is not based on insulin (41). The study demonstrated that the combined use of smoking and alcohol was associated with a higher average TyG index compared to smoking or alcohol consumption alone (42).

There are no reports indicating that the combined use of alcohol consumption and smoking directly diminishes the effectiveness of SNAC. However, the individual adverse effects of alcohol and smoking, such as further reduction in gastric pH and impaired gastrointestinal motility, could plausibly explain the diminished efficacy of oral semaglutide. According to a systematic review reporting the association between smoking, alcohol consumption, and functional gastrointestinal disorders (FGID), smoking has been linked to functional dyspepsia (FD), one of the most common FGIDs. Smokers were found to have a 50% higher risk of developing FD compared to non-smokers (43). On the other hand, moderate alcohol consumption does not appear to be associated with FGIDs, but excessive alcohol intake has been suggested to contribute to the onset and exacerbation of FGID symptoms, particularly FD (43). In this study, although the quantity of alcohol consumption was not evaluated, it is possible that among smokers, those who engaged in heavy drinking experienced functional dyspepsia (FD) and were unable to achieve the desired efficacy of oral semaglutide.

There are several limitations to the study that should be noted. First, the study included a small number of patients. Second, the 3 mg oral semaglutide dosage is used exclusively for dose escalation over a period of at least 4 weeks, as per official prescribing information and clinical trials, and is not recommended as a therapeutic maintenance dose. Third, the study did not evaluate key factors such as dietary habits, physical activity levels, and adherence to treatment, all of which could significantly impact the effectiveness of oral semaglutide therapy. Fourth, this study is subject to selection bias. Patients were enrolled only after completing the first 12 weeks of oral semaglutide treatment. Consequently, those who discontinued treatment during this initial period, for example, due to gastrointestinal adverse events, were not enrolled. This selection may lead to an overestimation of tolerability and limit the generalizability of the efficacy findings to all patients initiating oral semaglutide. Finally, as the study only involved a survey on the current history of alcohol drinking and smoking, it may have included some “occasional alcohol drinkers or smokers” among those who reported a history of alcohol drinking or smoking. Therefore, the findings of this study require validation through a larger-scale investigation using a more detailed questionnaire that addresses both alcohol drinking and smoking histories. We are planning to conduct large-scale studies that will include detailed information on the type, quantity, and frequency of alcohol consumption (e.g., weekly drinking frequency and per-event consumption), as well as smoking behaviors, such as the number of cigarettes smoked and the duration of smoking history.

Despite these limitations, this study was able to identify “no current history of either smoking or alcohol drinking” and “taking 31 minutes or longer to have breakfast after drug administration” as important factors that enhance the effect of oral semaglutide, thereby providing valuable insights into how the drug can be used more effectively in future clinical practice.

## Data Availability

The datasets presented in this study can be found in online repositories. The names of the repository/repositories and accession number(s) can be found in the article material.
