# Correction: Exploring factors predicting the effectiveness of oral semaglutide in Japanese individuals with type 2 diabetes switching from dipeptidyl peptidase 4 inhibitors: a pilot study

**DOI:** 10.3389/fcdhc.2026.1914250

**Published:** 2026-07-06

**Authors:** Takao Hirotsu, Kanta Taniguchi, Rimei Nishimura

**Affiliations:** 1 Department of Diabetes, Endocrinology and Hematology, Fuji Municipal Central Hospital, Fuji, Japan; 2 Department of Internal Medicine, Taniguchi Medical Clinic, Fujinomiya, Japan; 3 Division of Diabetes, Metabolism and Endocrinology, Department of Internal Medicine, Jikei University School of Medicine, Minato, Japan

**Keywords:** alcohol drinking, DPP-4 inhibitors, GLP-1 receptor agonists, type 2 diabetes, smoking

In the published article, one of the eligibility criteria for the patients included in the study was not stated with the same level of detail as in the IRB-approved protocol.

A correction has been made to the **Methods** section, subsection *Patients*, paragraph 1:

“iii) those who had previously received DPP-4 inhibitors and were deemed by their treating physicians to require transition to a GLP-1 receptor agonist, subsequently initiated oral semaglutide at a starting dose of 3 mg, and, at an outpatient follow-up visit conducted at least 12 weeks after initiation, had no adherence- or tolerability-related barriers to continued treatment;”

The original version of this article has been updated.

